# Supplementary material for: EPS364, a Novel Deep-Sea Bacterial Exopolysaccharide, Inhibits Liver Cancer Cell Growth and Adhesion
Source: Mar Drugs. 2021 Mar 22;19(3):171. doi: 10.3390/md19030171 (PMC8004136; doi:10.3390/md19030171)
Supplement: Supplementary file 1 [file marinedrugs-19-00171-s001.pdf]

Table S1. Primers used for the quantitative real-time PCR amplification

Primer	Sequence (from 5' to 3')
ALCAM-F	CCTTGCCGACTTGACGTACC
ALCAM-R	TGCCTCAAACACGTTGTCCT
ICAM1-F	GAACCCATTGCCCGAGCTCA
ICAM1-R	TGACAGTCACTGATTCCCCGAT
CTNNB1-F	TGTTAAATTCTTGGTATTACGACA
CTNNB1-R	CCACCACTAGCCAGTATGATGA
CAV-1-F	TTCTGGGCTTCATCTGGCAAC
CAV-1-R	GCTCAGCCCTATTGGTCCACTTTA
CAV-2-F	CACCCTCAGCTGTCTGCACAT
CAV-2-R	GGCAGAACCATTAGGCAGGTCTT
GAPDH-F	GCACCGTCAAGGCTGAGAAC
GAPDH-R	TGGTGAAGACGCCAGTGGA
CDH2-F	CGAATGGATGAAAGACCCATCC
CDH2-R	GCCACTGCCTTCATAGTCAAACACT
ALP-F	AGCTTGGTGGTGGATGAAAC
ALP-R	TCTTGCTTCATCGTTTGCAG
KLB-F	GCAGTCAGACCCAAGAAAATACAGA
KLB-R	CCCAGGAATATCAGTGGTTTCTTC
FGFR4-F	TCAAGCTCATCCCTGGTACG
FGFR4-R	GCCACAGTGCTGGCTTGGTCAG
FGFR19-F	GGAGATCCGCCCAGATGGCTAC
FGFR19-R	GGCCTCCAGTCCGGTGACAAGC
